# Phenology, mobility and behaviour of the arcto-alpine species *Boloria napaea* in its arctic habitat

**DOI:** 10.1038/s41598-019-40508-7

**Published:** 2019-03-07

**Authors:** Stefan Ehl, Stephanie I. J. Holzhauer, Nils Ryrholm, Thomas Schmitt

**Affiliations:** 1 Biogeography, Trier University, D-54286 Trier, Germany; 2 Senckenberg German Entomological Institute, D-15374 Müncheberg, Germany; 3 Leibniz Centre for Agricultural Landscape Research (ZALF), D-15374 Müncheberg, Germany; 4 Department of Electronics, Mathematics and Natural Sciences, University of Gävle, SE-80176 Gävle, Sweden; 5 Entomology, Department of Zoology, Institute of Biology, Faculty of Natural Sciences I, Martin Luther University Halle-Wittenberg, D-06099 Halle (Saale), Germany

## Abstract

Arctic and alpine environments present extreme, but different, challenges to survival. We therefore studied the ecological adaptation of the arctic-alpine fritillary *Boloria napaea* in northern Sweden and compared these results with the eastern Alps. Using mark-release-recapture, we analysed phenology, mobility, activity patterns, change in wing condition and nectar sources. The phenology showed no protandry, but a longer flight period of the females. Wing conditions revealed a linear decay being quicker in males than females. The mean flight distances were higher for males than females (143 vs 92 m). In general, males were more flight active, while females invested more time in feeding and resting. The shortness of the flight period in the Arctic is apparently a particular adaptation to these harsh conditions, not even allowing protandry, and constraining all individuals to hatch during a short period. These conditions also forced the individuals to concentrate on flight and alimentation. In general, Arctic and Alpine populations of *B. napaea* show few differences, but the species seems to be even better adapted to the northern environments. Thus, the short temporal separation of these populations seems not to have been sufficient for a divergent adaptation in the southern mountains.

## Introduction

Arctic and high mountain ecosystems in Europe share habitats with rather harsh conditions^1,2^. Although these habitats are often regarded as very similar, each ecosystem has its own special conditions, which may lead to different forms of adaptations, even in a single species^3^. Compared to the high mountain systems in the southern part of Europe, the climatic conditions affecting survival are probably even more extreme in the arctic realm: short and cold summers follow long, dark winters, while the intensity of insolation is much lower, and the sources of warm air and summer anticyclones are much further away, which leads to fewer flower-rich grassland on southern slopes for example^4^. This results in a much greater instability of the arctic summer, with suitable conditions for activity of some adult insects often restricted to a few days. Consequently, strategies well suited to survival in the southern high mountain systems might not be sufficient for survival in the Arctic, where additional challenges may have to be faced. Family-level studies on arctic arthropods support this hypothesis, and suggest that the timing of snowmelt and fluctuating temperatures may have an important effect on phenological variation, which is triggered solely by the prevailing conditions in the respective year^5–7^.

However, despite these differences between arctic and high mountain ecosystems, the occurrence of identical species in both of them is frequent, although taxonomic divergence is still taking place^8^. These so-called arcto-alpine disjunctions are often based on common ancestry with highly similar genetic structures found in several species^9–12^. Such large geographic disjunctions are mostly the result of wide, zonal ice age distributions in the periglacial steppe regions during glacial periods, followed by postglacial retreats northwards and uphill^8,13^. Therefore, a species’ characteristic traits in both parts of such a disjunct distribution must be seen in the light of its recent (i.e. postglacial) adaptations to the respective diverging habitat conditions. Consequently, comparative studies of the ecology of arctic and high mountain populations of such species are of high interest with regard to the adaptation of ecological traits within a single species over comparatively short periods of time (i.e. the postglacial).

Butterflies are one of the groups well-suited for ecological surveys^14,15^. However, although arcto-alpine disjunctions are common in some taxonomic groups^8^, they are rare in the European butterflies. Only three such species are known, i.e. *Boloria napaea* (Hoffmannsegg, 1804), *Erebia pandrose*, and *Pyrgus andromedae*^16^. So far, none of these species has been simultaneously studied in the southern alpine habitats and the arctic tundra using Mark-Release-Recapture (MRR) to characterise and compare their phenology, demography, behaviour, and mobility in these two parts of their range.

Here, we accordingly present a study on *Boloria napaea* (Hoffmannsegg, 1804) in its arctic habitat, thus adding the northern perspective to the already existing Alpine study^17,18^. In particular, we ask whether the phenological, dispersal, and behavioural traits of *B. napaea* are comparable in both regions, permitting a better understanding of the effects of these different environments on the evolution of different ecological traits. We expect that, if conditions in the arctic and the alpine environment have shaped *B. napaea* similarly, arctic populations also show (i) no asynchrony in emergence of the sexes, and (ii) no prolonged emergence period, but (iii) patterns of mobility similar to alpine ones. If this is the case, the species can be regarded as adapted to both arctic and alpine conditions, but not particularly well to either. However, the traits so far observed in the Alps might also be regarded more as adaptations to the Arctic realm, which have not been modified in the Alps, perhaps simply because the comparatively short duration of the postglacial has been insufficient to allow adaptation to the rather different conditions. On the other hand, if arctic *B. napaea* populations should show adaptations very similar to the ones observed for alpine butterfly species such as *B. pales* (e.g. protandry of males and a prolonged emergence period^17^), and thus different traits than in the Alps, this could indicate a generally stronger adaptation to arctic than to alpine conditions. This scenario would speak for a rapid (and consequently imperfect) adaptation to high mountain ecosystems. Such a finding would be consistent with the characterisation of this species as a mostly arctic element, rather than a high mountain element. In this study, we test these alternative hypotheses, and simultaneously enlarge the knowledge on adaptation strategies of arthropods to arctic conditions.

## Results

### Capturing data

From 20 July to 15 August 2016, we marked 261 individuals (170 males; 91 females) and obtained recaptures of 59 individuals (45 males; 14 females). We accomplished one recaptures event for 32 males and 13 females, as well as multiple recaptures for both sexes: 6 males were recaptured twice, 6 males and 1 female were recaptured three times (for the distribution of the capture events see Supplementary Fig. S1).

### Population demography

We chose for further analyses the model with the lowest Akaike Information Criterion (AIC_C_) and the smallest number of parameters (Table 1). Models with a difference between them in AIC_C_ of <2 have equal weights of data, as outlined by Burnham & Anderson (2002)^19^, and therefore, we also included in our selection as a further criterion, the number of parameters used by the models. Under these conditions, the best model presumed an additive dependency of sex and linear time on the survival rate, an interactive dependency of sex and sampling effort on the capture probability, a time in factorial dependency on the proportional recruitment, and a no dependency number of individuals in the population.

**Table 1:** Akaike information criterion (AIC_C_) and number of considered parameters of the three best models for estimating the daily population sizes of *B. napaea* in 2016 at Nuolja Mountain in the Abisko National Park, Sweden with POPAN 5.0; the model with the best combination of low AIC_C_ and small number of parameters (second model) was chosen as best supported.

Model	AICc	Parameters
{*Phi*(*g* + *T*) *p*(*g* x *hours*) *pent*(*g* + *t*) *N*(*g*)}	613.80	14
{*Phi*(*g* + *T*) *p*(*g* x *hours*) *pent*(*t*) *N*(.)}	614.01	13
{*Phi*(*g* + *T*) *p*(*g* x *hours*) *pent*(*g* + *t*) *N*(.)}	614.75	14

Basic variables: survival rate (*phi*), capture probability (*p*), proportional recruitment (*pent*), total number of individuals (*N*). Dependent variables: sex (*g*); factorial (*t*), and linear (*T*) dependency on time; no dependency (.).

This model estimated a total number of 434 male individuals (±42 SE), i.e. 76 individuals per hectare (±7.4 SE), and 291 females (±35 SE), i.e. 51 individuals per hectare (±6.1 SE). Hence, the proportion of females was only about 67% of the males. In total, this amounts to 127 individuals per hectare and a total population size of 725 individuals. The subsequently following models yielded similar results in terms of census size and general phenology.

**Figure 1:**
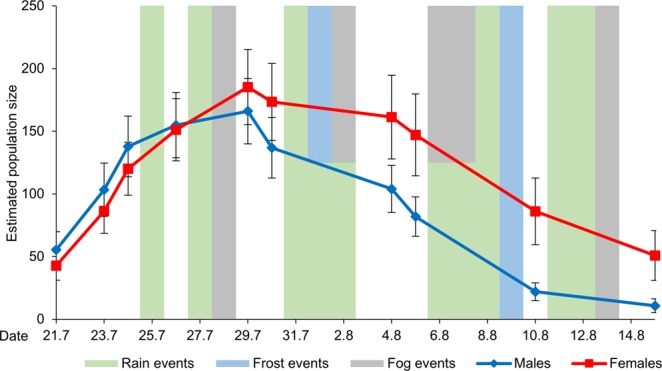
Estimated population size of *B. napaea* for every sampling day. Vertical bars represent days with extreme weather events (green bar: no sampling on days with rain events, light blue bars: no sampling on days with frost events, grey bars: no sampling on days with fog events; bars with two colours represent days with two extreme weather events); error bars represent the standard error of the calculated population size from the program MARK.

From the beginning of the flight period, the daily number of individuals of both sexes increased rapidly, but the number of females increased with a delay of roughly two days. After 26 July, when the estimates for both sexes reached more or less the same size, the number of females exceeded the number of males until the end of the flight period, i.e. 15 August. The population decreased after 29 July, with the decline being less pronounced in females than in males (Fig. 1).

### Wing condition

**Figure 2:**
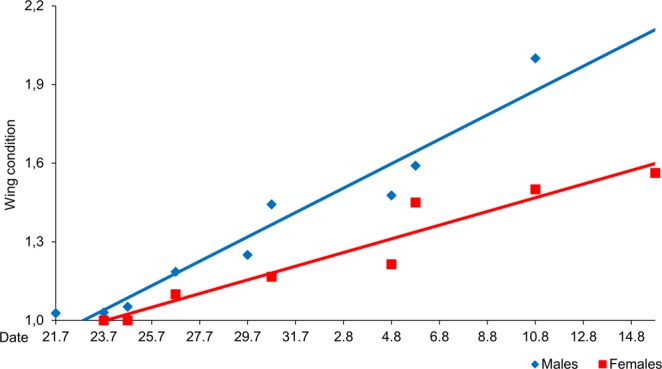
Age structure of *B. napaea* computed from the daily wing condition. Blue trend line: linear function for males, red trend line: linear function for females; daily mean values males as blue rhombuses, females as red squares.

Average wing conditions based on at least five individuals per day and sex were calculated for nine days for males and for eight days for females (Fig. 2). The equations derived from linear regression for both sexes showed similar results (males: y = 0.04653 x − 2,204.4*, R²*_*adj*_ = 0.93; females: y = 0.0261 x − 1,130.1, *R²*_*adj*_ = 0.92). For both sexes a constant deterioration of the wing conditions over the entire flight period (without an increasing towards the end) was observed. However, the regression line for the males had a steeper slope and a smaller x-intercept than the line representing the female population. Hence, both lines did not run in parallel over the flight period. This difference was due to the faster deterioration of wings (∆ wing condition) of the males (0.10 per day ±0.01 SE) than of the females (0.09 per day ±0.01 SE), especially towards the end of the flight period. Both linear regressions were highly significant (males: F = 107.32, *P* < 0.001; females: F = 76.90, *P* < 0.001).

### Mobility and movement patterns

**Figure 3:**
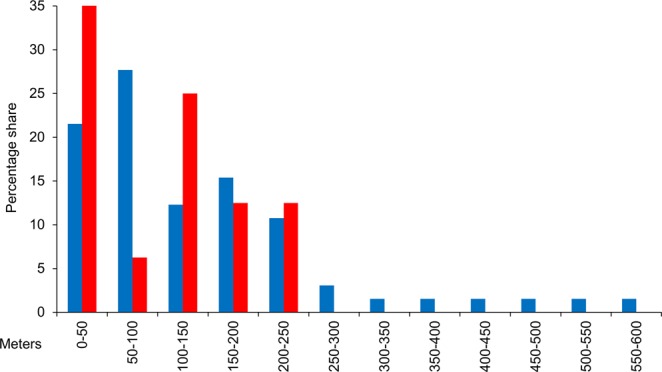
Percentage of recaptured individuals of *B. napaea* in combination with their movement distances between capture and first recapture event. The distances are divided into 50 m intervals; blue bars represent males, red bars represent females.

With a mean dispersal distance of 142.6 m ±15.6 SE (n = 65), male individuals moved larger distances than females with a mean dispersal distance of 91.7 m ±17.6 (n = 16), although these differences were not significant (U test: *P* = 0.14). The maximal recorded distance of males (559 m) was also larger than for females (212 m) (Fig. 3). We fitted the inverse cumulative proportion values of individuals moving certain distance classes to the negative exponential function (NEF) and the inverse power function (IPF) and compared the adjusted stability indices (i.e. *R²*_*adj*_); the 50 m intervals of both sexes showed the highest fitting. The resulting equations for NEF and IPF were highly significant (all *P* < 0.001). For both sexes, the fittings were better for NEF than for IPF, whereby NEF estimated considerably lower dispersal probabilities than IPF for all distances (Table 2). The proportion of individuals moving distances of 1 km, 2 km, 3 km, and 5 km, calculated with NEF and IPF (based on 50 m intervals) estimated higher dispersal probabilities for males than for females with NEF, whereas IPF predicted a higher proportion of female long-distance dispersal (Table 3). This, however, might be an artefact resulting from the noticeably smaller numbers of 50 m intervals that could be used for the calculations of female dispersal: While twelve 50 m intervals were included in the NEF and IPF calculations for males, only five intervals were useable for females. This leads to a lesser sloping of the curve-fittings and a calculation of a higher long-distance dispersal rate. According to NEF, only one male specimen of 1,000 and about one female specimen in 10,000 would cover 1 km. In contrast, IPF estimated more than one male in a hundred for the same distance class and one female in a hundred for 3 km.

**Table 2:** Adjusted stability Index (*R²*_*adj*_) for IPF and NEF calculated with movement distances of *B. napaea* (based on 20 m, 30 m and 50 m intervals). Underlined numbers represent the highest values for the adjusted Coefficient of determination (*R²*_*adj*_).

	20 m intervals		30 m intervals		50 intervals	
	IPF	NEF	IPF	NEF	IPF	NEF
Males	0.88	0.97	0.89	0.98	0.89	0.99
Females	0.76	0.92	0.72	0.91	0.84	0.95

**Table 3:** Calculated percentage of individuals from *B. napaea* which would move 1 km, 2 km, 3 km or 5 km (calculated with IPF and NEF based on 50 m intervals).

Distance	Interval Number	IPF Males	NEF Males	IPF Females	NEF Females
1 km	20	1.50	0.10	3.54	0.01
2 km	40	0.47	6..44^−5^	1.57	4.00^−7^
3 km	60	0.24	4.27^−8^	0.98	1.93^−11^
5 km	100	0.10	1.87^−14^	0.54	4.49^−20^

### Behavioural differences between sexes and nectar preferences

χ²-homogeneity tests revealed sexual dimorphism in behaviour (χ² = 49.9, *df* = 3, *P* < 0.001; n = 271). Here, male individuals showed a higher flight activity (♂♂: 70.8% vs. ♀♀: 38.1%), whereas females were observed feeding (♂♂: 11.9% vs. ♀♀: 29.5%) or resting (♂♂: 11.4% vs. ♀♀: 32.4%) more frequently. Interaction was only observed in male individuals, but this did not form a large component of their behaviour (♂♂: 5.9% vs. ♀♀: 0.0%).

The selection of inflorescences for nectaring (n = 59 visits: males 28, females 31), assessed at plant family and genera level, revealed no sex-specific difference (χ²-homogenity-test: plant family: χ² = 2.3, *df* = 3, *P* = 0.52; plant genera: χ²-homogenity-test: χ² = 6.2, *df* = 5, *P* = 0.29). Hence, pooled data for both sexes was used to compare the observed and expected nectar sources (from inventories of plant communities and the assumption of random flower-use). Only Asteraceae (54 of 59 visits) were used more frequently than expected, whereas all other plant families with observed visits (14 plant families without visits) were used as expected (Caryophyllaceae and Rosaceae) or even less (Ranuculaceae) (Table 4). Subsequently, we only performed further analysis within the family Asteraceae, where Jacobs’ index and Bailey’s confidence intervals revealed that the genus *Taraxacum* was the only one more frequently visited than expected (50 visits), while the other genera of the Asteraceae were used as expected (1 visit *Erigeron*, 3 visits *Saussurea*) (Table 4).

**Table 4:** Selection of nectar sources from *Boloria napaea* (sexes pooled) using Jacobs’ index of selection (Jacobs 1974; classification for our study: 1 to 0.33 preference, 0.33 to −0.33 neutrality, −0.33 to −1 avoidance) and Bailey’s confidence intervals at *P* value < 0.05 (Bailey, 1980); rating: “+” preference nectar source, “=” neutrality, “−“ avoidance; n is the total number of observed plant families respectively genera during the study period; on plant genera level, only genera from the family Asteracea were observed.

	Category	Observed visits	Proportion expected	Proportion used	Jacobs‘ index	Jacobs‘ index rating	Bailey’s confidence intervals	Bailey’s confidence intervals rating
Plant families; n = 18	Asteraceae	54	0.265	0.915	0.94	+	(0.701;0.982)	+
	Caryophyllaceae	3	0.060	0.051	−0.09	=	(0.000;0.200)	=
	Ranunculaceae	1	0.220	0.017	−0.88	—	(0.008;0.142)	—
	Rosaceae	1	0.070	0.017	−0.63	—	(0.008;0.142)	=
Plant genera; n = 28	*Erigeron*	1	0.025	0.017	−0.20	=	(0.008;0.142)	=
	*Saussurea*	3	0.045	0.051	0.06	=	(0.000;0.200)	=
	*Taraxacum*	50	0.070	0.847	0.97	+	(0.702;0.983)	+

## Discussion

The phenology, mobility and dispersal pattern as well as the behaviour of arctic *B. napaea* in general showed remarkable similarity with alpine populations. However, in the northern part of its range, the species seems to be additionally adapted to the even shorter suitable periods by the duration of the flight period and the greater time invested in feeding by females.

Protandry and sequential emergence are seen as adaptations to the length of the vegetation period and the unpredictability of the prevailing conditions and can be highly beneficial for the maintenance of populations^20–22^. Consequently, many lowland butterfly populations use the advantages that protandry and emergence over a short period of time offer^23–25^. However, extreme climatic conditions challenge the advantage of protandry. Thus, no protandry has been found for *Euphydryas aurinia glaciegenita*^26^ (the Alpine subspecies of *E. aurinia*^27^) belonging to a species widely distributed in the lowlands of the Palaearctic, where it mostly exhibits a typically protandrous structure^28–31^. So, avoiding protandry might be a strategy of taxa that are not perfectly adapted to an extreme environment.

However, some typical high mountain species are showing only partial protandry, e.g. the high mountain specialist *Erebia nivalis*, an Alpine endemic^32^. As this species is one of the butterflies adapted to the highest elevations^33^, one might argue that the harsh conditions in these areas are not compatible with the establishment of full protandry, which is highly favourable under lowland conditions. Full protandry, nevertheless, also exists in high mountain species such as *Boloria pales*, a close relative of our study species^34^. By combining protandry with sequential emergence, this species spreads the risk of prolonged periods of bad weather throughout the flight period^17^. In contrast, neither the arctic nor the high mountain population of *B. napaea* exhibited protandry, although males of both populations decreased more strongly than females towards the end of the flight period, starting two weeks before its respective end^17^.

In our arctic study area, however, the flight period of *B. napaea* lasted only 26 days. Hence, the Alpine *B. napaea* population studied previously, differed considerably from the northern population in this respect (68 days in 2012)^17^. Consequently, the complete lack of protandry of *B. napaea* in the arctic area might also be a result of the much shorter flight period, which starts later and ends sooner. However, the onset of the flight period might differ considerably from year to year in the Abisko region with sightings even in late June and early July (see Link: https://www.artportalen.se/search/map/taxon/201073) underlining the strong correlation with the prevailing weather conditions of the respective year. Due to these early sightings, inter-annual variation in the phenology of the species has to be assumed, and further studies are necessary to corroborate the length and starting date of the flight period over the years. Nevertheless, even with the earliest start possible, a duration of about 40 days might put the species under higher pressure to adapt to a shorter flight period than in the Alps, and consequently leads to synchronised emergence of males and females.

As already mentioned above, constant emergence rates over most of the flight period represent another adaptation strategy to harsh and unpredictable climatic conditions by spreading the risk of mortality caused by long-lasting bad weather conditions. This strategy is known for example for the alpine *B. pales*, but was observed much less for *B. napaea* in the Alps^17^ and was absent in our present study in the Arctic as indicated by the proportion of bad wing conditions rapidly increasing towards the end of the flight period in both sexes (i.e. no freshly emerged specimens can mask the constant wing decay of the existing individuals, which led to a constant wing deterioration over the entire flight period without an increasing deterioration at the end).

Furthermore, *B. napaea* was more sensitive to snow and frost events than *B. pales*, which is reflected by the higher population size variation and lower survival probability in the former^17^. In the case of *B. napaea*, we therefore assume that the lack of a protandrous demographic structure with the majority of males and females hatching synchronously, is the effect of mostly being adapted to the specific challenges arising from arctic conditions with the shorter “window” for the flight period and even more unpredictable weather. We therefore postulate that, even originally, *B. napaea* was not protandrous, and that so far this has not changed under the specific conditions in the Alps.

Female butterflies in general invested more in gaining and keeping resources (and hence in egg production) while males spent more time and hence resources in flying (e.g. in patrolling behaviour searching for females and hereby optimising their reproductive success)^23,24,35–37^. Male flight activity in our study on *B. napaea* in the Arctic, where males spent more than two third of the observed time flying and only rarely rested, was even more pronounced than in the eastern Alps. The much higher proportion of flying males than females (twice as many; 1.5 in the eastern Alps) and also their slightly longer flight distances (as e.g. observed for the cumulative flight distances) can therefore explain the faster decay of the males’ wings in our study. Consequently, males in the north were resting for only one third of the time observed in the Alps, while females in the arctic study area seemed to rest as often as females and males in the Alps. This interregional difference in behaviour was not at the expense of feeding, as both the proportion and the ratio of feeding males and females were very similar in both areas (1:3). This strong focus of arctic *B. napaea* males on flying and feeding might be one of the species’ adaptations to the very short flight period and the high environmental unpredictability in the Arctic if compared with alpine conditions.

Despite the pronounced behavioural differences, both sexes in the Arctic used *Taraxacum* as the nearly exclusive source of alimentation. This was in strong contrast to the eastern Alps, where the sexes differed remarkably in their flower selection^18^. Such differences are often explained by different nectar compositions offered by different plant species with differing amounts of sugar (necessary for flight) and amino acids (necessary for egg production)^38–40^. The nearly exclusive use of *Taraxacum* by both sexes in our arctic study area suggests that the nectar has a balanced content of sugar and amino acids suitable for the alimentation of both sexes. However, testing this hypothesis requires investigation of the nectar composition of arctic *Taraxacum*.

The general mobility patterns observed in the present study performed in the Arctic were concordant with the ones observed for *B. napaea* in the eastern Alps^18^. Thus, distances between sexes did not differ significantly and the large majority of males and females did not move beyond 200 m (mean distances Arctic vs. eastern Alps: 143 m ±15.6 SE and 123 ± 7.9 SE, respectively, for males, and 92 m ±17.6 SE and 105 m ±8.5 SE, respectively, for females). Maximum distances of a few male individuals were up to ca. 600 m in both studies. Consequently, the size of the study areas (5.7 vs. 29.4 ha) seemed not to affect these distances. In comparison with other alpine butterfly species, *B. napaea* showed a lower mobility within its habitat than *B. pales*^18^, but was similarly mobile as *E. nivalis*^32^. Nevertheless, individuals of the Alpine subspecies *Euphydryas aurinia glaciegenita* even exhibited a considerably more sedentary behaviour^26^.

However, the estimation of dispersal rates and dispersal itself might also depend strongly on population density^41,42^. Estimated numbers of individuals for the arctic study area amount to 127/ha. This is a little less but nevertheless comparable to the 163/ha estimated for the less suitable part of the study area in the eastern Alps^17^. However, the density in the more suitable part of the analysed area in the latter study (563 females/ha; male density could not be estimated but should have been of a similar order of magnitude) suggests that the rather low observed density of *B. napaea* in our arctic study site was either the result of generally lower population densities in these arctic environments, or that our study was performed during a year with a low population size. However, the abundance of *B. napaea* also may have been influenced by host plant density and spatial patch isolation^43–45^. Furthermore, and in contrast to its vicinity, the arctic flower-rich meadow provided an abundance of nectar plants at the time of our study, which may have had a remarkable isolating affect. This and the low local population size might also explain the generally low dispersal activity in our study in the Arctic.

According to our estimations, *B. napaea* is also a relatively poor long-distance disperser. This holds true for the Arctic (this study) and the Alpine area^18^. In both areas and independently from the mathematical model used, only a small fraction of both sexes disperses further than 1 km. While the NEF model (supported by higher adjusted *R*^2^_*adj*_ than the IPF model) calls for very limited long-distance dispersal, IPF supports a moderately higher rate of dispersal with long distance movements mostly by females in the arctic areas. Particularly the results obtained using the NEF model make ecological sense for the arctic population studied here, due to high selective pressures outside the studied patch: No other patch of high quality habitat occurs in the surrounding landscape so that individuals are favoured which invest more in reproduction in their patch of origin than in dispersal beyond it^46^. Considering the low population density in our study year, the carrying capacity might have been sufficient to prevent individuals being forced to emigrate.

At least, the situation in the eastern Alps with many suitable habitats scattered around the surveyed area might be different. Baguette (2003) has already observed that the NEF model (even when showing higher adjusted *R*^2^_*adj*_ values) often yields results inferior to those of the IPF model in accounting for long distance movements^47^. In this context, the higher rates of long distance dispersal calculated for females compared with males, for both survey regions, is ecologically meaningful, because finding empty high habitat quality patches beyond the borders of the habitat of origin is highly beneficial for females, but useless for males^18^. Hence, a decision on which of the two models is more appropriate for the arctic population of *B. napaea* needs additional research.

In comparison to its area of alpine distribution, *B. napaea* in the northern areas seems to be even more closely adapted to the short vegetation period and the utilisation of food sources. However, variance of weather conditions from year to year renders it necessary to further study this arcto-alpine butterfly species. Currently, direct and indirect effects of climatic changes on the distribution of butterflies (like longer seasons or timing of the snowmelt) cannot be assessed, although our knowledge about this species has improved, but is still not sufficient for answering these questions. However, negative effects on *B. napaea* are expected because of its future climate driven range losses that have to be expected as in other related species of the *Boloria*/*Clossiana*/*Proclossiana* complex with arctic distribution areas^48^. In addition, conservation strategies developed for alpine or boreo-montane species might not be applicable to arcto-alpine butterfly species, and the possibility to avoid the consequences of climatic change by shifting to higher or more northern areas is limited in the actual distribution area of *B. napaea* in the European Arctic^49^.

## Materials and Methods

### Mark-release-recapture (MRR)

From 20 July to 15 August 2016, we performed a MRR study at Nuolja Mountain in the Abisko National Park (68°22′N, 18°43′E) with *Boloria napaea* (Red list: least concern^50^). We surveyed over the entire flight period of the respective year. From 14 to 19 July, weather conditions were bad, and no specimens were observed. Although, the study area had no hard borders (north: a larger snowfield; south: Aurora Sky station with the network of rambling trails; west: steep slopes nearly without vegetation; east: beginning of shrubland from lower regions), they enclosed an area with a comparable presetting (for example nectar sources) to the one in the Alps. Outside of this area no larger patches with a comparable density of flowering plants or an acceptable number of butterflies (to perform a study) were found. To weaken the problem of the soft borders, we controlled the surroundings of the area for marked butterflies each day at the beginning and or at the end of our survey. During the flight period, only ten days offered suitable weather conditions for butterfly activity (weak or moderate wind, no rain, frost, fog or snow). Every newly captured butterfly was marked with a fineliner on the bottom side of the wings, without harming it. Prior to release of the specimen, the following data were recorded: sex, GPS position of capture point, capture time, wing wear (score: 1: wing fringe completely undamaged; 2: wing fringe is missing but wings are still undamaged; 3: wings slightly damaged; 4: wings seriously damaged^30,37,51^), behaviour (i.e. flying, resting, feeding, interaction) and, if applicable, the nectar source species visited (same information were recorded for all recaptures). To detect sexual dimorphism in behaviour, we performed χ²-homogeneity tests based on the four recorded behavioural categories (only first captures were used to avoid individual preferences). Average wing conditions for both sexes were calculated for each day. With these data, we calculated the degradation of wings (∆ wing condition) for both sexes over time in order to obtain further information on the demography of the population. After setting the wing condition and the day of the first capture to zero, we computed linear regressions on the basis of differences between wing conditions on the first and following capture days. All univariate statistics were calculated in SPSS 22.0^52^.

We used the module POPAN 5.0^53^ in the program package MARK 8.0 to estimate daily population sizes based on the Jolly-Seber method for open populations^54^. We estimated population sizes separately for both sexes. POPAN 5.0 estimates three primary parameters: survival probability (*Phi*), capture probability (*p*) and proportional recruitment (*pent*). These parameters may be constant (.), dependent on sex (*g*), respond to time in factorial (*t*) or linear (*T*) manner, or display additive (*g* + *t*, *g* + *T*) or interactive (*g* * *t*, *g* * *T*) interactions^29,55,56^. The capture probability might also depend on the sampling effort (hours)^53^. After a Goodness-of-Fit Test (option: RELEASE), we analysed different combinations of the parameters mentioned above. We selected as best supported the model with the lowest value for the corrected Akaike Information Criterion (AIC_C_)^57,58^ and the smallest numbers of parameters^19^.

### Mobility and movement patterns

To analyse the movement behaviour of *Boloria napaea*, we measured the direct distance travelled from one to the next capture event for all individuals with ArcGIS 10.2.1^59^. With these data, we performed a Mann-Whitney U test to reveal possible differences in mobility between sexes.

Afterwards, we used the computed single distances to calculate the inverse cumulative proportion of individuals moving in certain distance classes, with each class representing an interval of 20 m (separately for both sexes). These data were fitted against two different mathematical models, to find the best prediction of long-distance movements^24,29,37,47,60^: the negative exponential function (NEF) and the inverse power function (IPF). Analyses were repeated with 30 m and 50 m intervals to exclude possible artefacts based on the 20 m interval size. Significance of the curve fittings for both mathematical models, was tested by performing F statistics in SPSS 22.0^52^. With the calculated adjusted stability Index *R²*_*adj*_ for the resulting curves, we chose the best model and interval size to predict the proportion of individuals moving greater distances than those covered by our MRR study^29,37,47^.

### Nectar sources

We performed three plant sociological inventories, one every ten days, following the method of Braun-Blanquet (1964)^61^ to assess all occurring plant species and their abundance during the study period^62^. To reveal possible differences in preferred nectar sources between sexes, χ²-homogeneity test were used. With the Jacobs’ index of selection^63^ and Bailey’s confidence intervals^64^ at *P* values < 0.05 we tested for differences in preferred nectar sources on the plant family and the plant genera level.

## Supplementary information

Supplementary Information for <i>Boloria napaea</i>

## Data Availability

The datasets used and/or analysed during the current study are available from the corresponding author on reasonable request. All data generated or analysed during this study are included in this published article [and its supplementary information files].
