# Supplementary material for: Phenology, mobility and behaviour of the arcto-alpine species Boloria napaea in its arctic habitat
Source: Sci Rep. 2019 Mar 7;9:3912. doi: 10.1038/s41598-019-40508-7 (PMC6405876; doi:10.1038/s41598-019-40508-7)
Supplement: Supplementary file 1 — Supplementary Information for Boloria napaea [file 41598_2019_40508_MOESM1_ESM.docx]

**SUPPLEMENTARY INFORMATION**

**Phenology, mobility and behaviour of the arcto-alpine species *Boloria napaea* in its arctic habitat**

Stefan Ehl*, Stephanie I. J. Holzhauer, Nils Ryrholm and Thomas Schmitt

*corresponding author:

Stefan Ehl, University of Trier, Universitätsring 15, D-54286 Trier, Germany

Email: ehlst@outlook.com

Tel.: +49-172-7479630

Tel.: +49-651-201 4691

Fax: +49-651-201 3851

**Methods**

Study species

*Boloria napaea* Hoffmannsegg, 1804 is a species with arctic-alpine disjunction and belongs to the family of Nymphalidae. In Europe, its geographically largest distribution is in Scandinavia. Here, the species is found from sea level to 1.100 m asl. In the Alps, it occurs from 1.500 to 2.500 m asl. ^1–3^. The species lives on flower-rich high-altitude grasslands and tundras; their larvae feed on *Viola* species and *Bistorta vivipara* ^1,2,4,5^. Usually, the flight period of *B. napaea* in the Alps starts in late June or early July and ends in early September, but the annual flight period strongly depends on the climatic conditions of the respective summer and the locality. However, the flight period in the northern habitats is often shorter ^1,2,4,6^. Normally, *B. napaea* occurs in one generation per year, and only in northern Scandinavia, the larval development may need two years ^2,5^. Due to the high abundance at its flight places (Huemer 2004), this species is a suitable model organism to analyse its population dynamics.

Study area

The mark-release-recapture (MRR) study site was located at Nuolja Mountain in the Abisko National Park (68°22´N, 18°43´E) near the Aurora Sky Station ([Norrbottens län](https://de.wikipedia.org/wiki/Norrbottens_l%C3%A4n), northern Sweden). It was a flower-rich meadow (size: 5.7 ha) situated on an east-facing hillside reaching from 800 to 980 m asl. Scattered flowering *Viola* specimens (one of the larval food plants) were sighted during the studied flight period of *B. napaea*. The Nuolja Mountain is a widespread area with similar but not equal habitats. Borders were: west: area with steep, rocky slopes nearly without plant cover; east: beginning of shrubland from lower regions of the mountain; north: there was a larger snowfield (even at the end of the study); south: Aurora Sky station with the network of rambling trails.

Further information Mark-Release-Recapture study

During these days, we sampled the entire study area, netting all available individuals. We marked every captured butterfly with an individual code consisting of a letter for the sampling day and a running number, always starting with “1” every day. Prior to release, the following data were recorded: sex, GPS position of capture point, capture time, wing wear (scored from 1 for fresh, to 4 for heavily damaged) ^7–9^, behaviour (i.e. flying, resting, feeding, interaction) and, if applicable, the nectar plant species visited. We also recorded this information for all recaptures. To avoid capture-release-trauma influencing our analyses ^10^, recaptures were not considered at the day of the capture event ^11^.

Equations for calculating negative exponential function (NEF) and the inverse power function (IPF)

For the NEF, the relative proportion of individuals moving to distance *D* is

*I_NEF_ = a*e*^-kD^* respective ln *I =* ln *a – kD*.

The parameter *a* represents a scaling constant while *k* is the dispersal constant describing the shape of the exponential curve. Under the IPF, the proportion *I* is expressed as

*I_IPF_ = cD^-n^* respective ln *I =* ln *c-n (*ln *D),*

where *c* is a scaling constant and *n* a variable describing the effect of the distance on dispersal ^12,13^.

**Supplementary Fig. S1:** Heatmap of *Boloria napaea* in Abisko (created with QGIS 2.18.15 ^14^); red line represents the border of the study area.

**
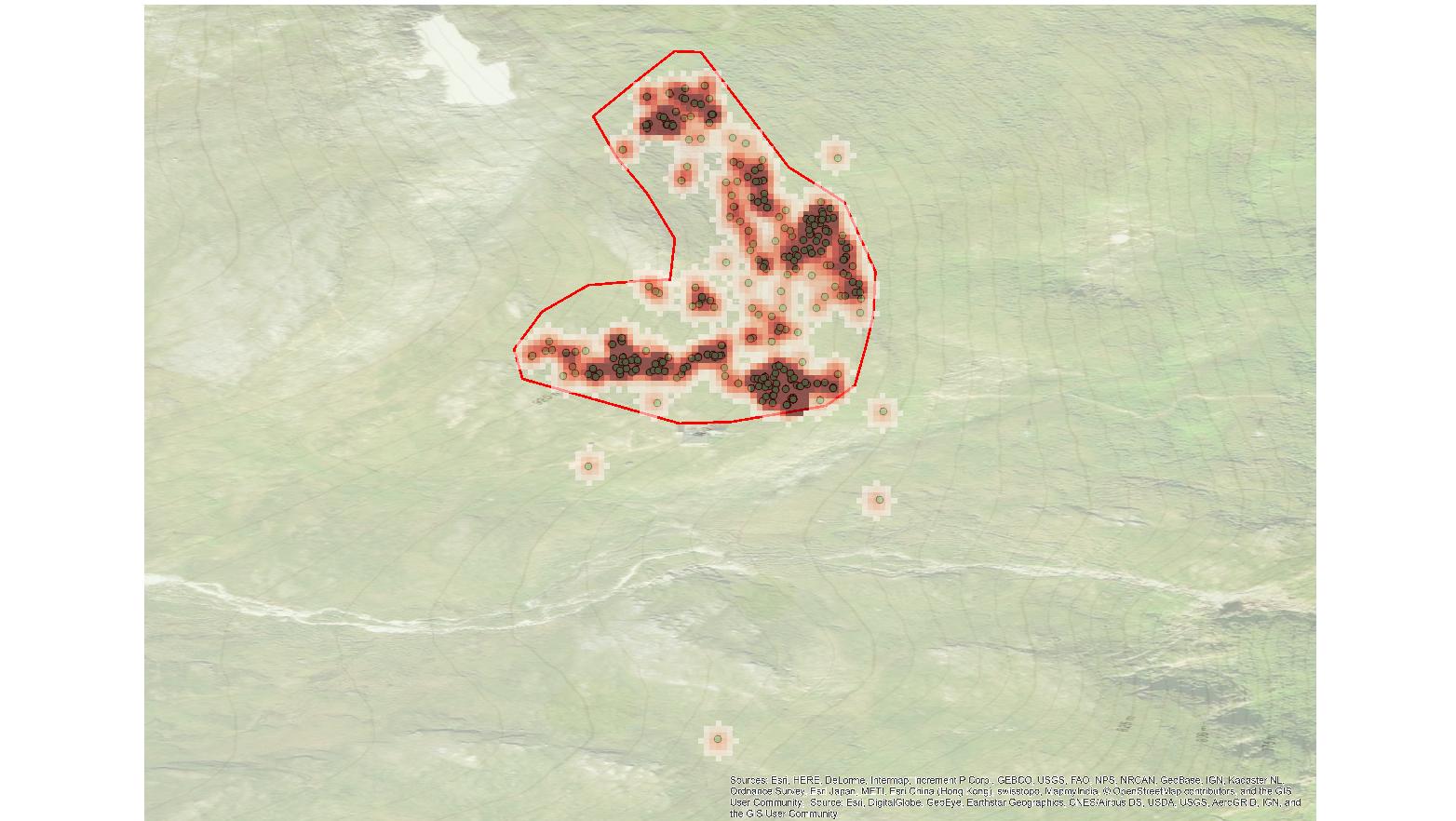
**

References

1. Tshikolovets, V. V. *Butterflies of Europe & the Mediterranean area* (Tshikolovets Publications, Pardubice, 2011).

2. Tolman, T. & Lewington, R. *Schmetterlinge Europas und Nordwestafrikas. Alle Tagfalter; über 2000 Arten.* 2nd ed. (Kosmos, Stuttgart, 2012).

3. Kudrna, O., Pennerstorfer, J. & Lux, K. *Distribution atlas of European butterflies and skippers* (Wissenschaftlicher Verlag Peks, Schwanfeld, 2015).

4. Schweizerischer Bund für Naturschutz. *Tagfalter und ihre Lebensräume. Arten, Gefährdung, Schutz ; Schweiz und angrenzende Gebiete.* 4th ed. (Schweizerischer Bund für Naturschutz, Basel, 1994).

5. Huemer, P. & Wieser, C. *Nationalpark Hohe Tauern. Schmetterlinge ; [wissenschaftliche Schriften* (Tyrolia, Innsbruck, Wien, 2008).

6. Thomas, J. A. The Ecology and Conservation of Lysandra bellargus (Lepidoptera: Lycaenidae) in Britain. *Journal of Applied Ecology* **20,** 59–83 (1983).

7. Zimmermann, K., Fric, Z., Filipova, L. & Konvička, M. Adult demography, dispersal and behaviour of Brenthis ino (Lepidoptera: Nymphalidae). how to be a successful wetland butterfly. *Eur. J. Entomol.* **102,** 699–706; 10.14411/eje.2005.100 (2005).

8. Thomas, J. A. The Ecology and Conservation of *Lysandra bellargus* (Lepidoptera: Lycaenidae) in Britain. *The Journal of Applied Ecology* **20,** 59; 10.2307/2403376 (1983).

9. Munguira, M. L., Martín, J., García-Barros, E. & Viejo, J. L. Use of space and resources in a Mediterranean population of the butterfly *Euphydryas aurinia*. *Acta Oecologica* **18,** 597–612; 10.1016/S1146-609X(97)80044-6 (1997).

10. Watt, W. B., Chew, F. S., Snyder, L. R. G., Watt, A. G. & Rothschild, D. E. Population structure of pierid butterflies. I. Numbers and movements of some montane Colias species. *Oecologia* **27,** 1–22; 10.1007/BF00345682 (1977).

11. Gall, L. F. The effects of capturing and marking on subsequent activity in Boloria acrocnema (Lepidoptera: Nymphalidae), with a comparison of different numerical models that estimate population size. *Biological Conservation* **28,** 139–154; 10.1016/0006-3207(84)90032-6 (1984).

12. Junker, M. & Schmitt, T. Demography, dispersal and movement pattern of Euphydryas aurinia (Lepidoptera: Nymphalidae) at the Iberian Peninsula:. an alarming example in an increasingly fragmented landscape? *J Insect Conserv* **14,** 237–246; 10.1007/s10841-009-9250-1 (2010).

13. Baguette, M. Long distance dispersal and landscape occupancy in a metapopulation of the cranberry fritillary butterfly. *Ecography* **26,** 153–160; 10.1034/j.1600-0587.2003.03364.x (2003).

14. QGIS Development Team. *QGIS Geographic Information System 2.18.15.* Available at https://qgis.org/ (2019).
